# High-frequency ultrasound combined with microbubbles for preoperative lymphatic mapping for lymphedema with a non-linear pattern in indocyanine green lymphography

**DOI:** 10.1007/s00330-025-12293-7

**Published:** 2026-01-24

**Authors:** ShuFang Yuan, LiPing Chen, LanJing Wu, ChenYang Zhao, JingJing WEN, Long Biao YU, ZheGang Zhou, DeSheng Sun, ZhengMing Hu

**Affiliations:** 1 Department of Ultrasound Imaging, Peking University Shenzhen Hospital, Shenzhen, China; 2 Medical Technology Department, The Islands Healthcare Complex—Macao Medical Center of Peking Union Medical College Hospital, Shenzhen, China; 3 Microsurgery & Lymphatic Surgery Department, Peking University Shenzhen Hospital, Shenzhen, China

**Keywords:** Lymphedema, Lymphovenous anastomosis, Ultrasonography, Contrast-enhanced ultrasound, Indocyanine green

## Abstract

**Objectives:**

Lymphatic-venous anastomosis (LVA) is an effective surgical treatment for lymphedema, which requires accurate identification of lymphatic vessels. Indocyanine Green (ICG) lymphography, the most common method for lymphatic mapping, cannot always successfully identify lymphatic vessels. We aimed to explore high-frequency ultrasound (HFUS) and contrast-enhanced ultrasound (CEUS) as a reliable alternative for lymphatic mapping when ICG lymphography is not feasible.

**Materials and methods:**

We performed combined HFUS and CEUS for lymphatic mapping on the patients who exhibited no obvious linear pattern on ICG lymphography. The inner and outer diameters and depths of the lymphatic vessels were measured. We subsequently evaluated the accuracy of US lymphatic mapping by comparing it with the operative results. And the postoperative volume and circumference of the affected limbs were compared with the preoperative measurements.

**Results:**

We recruited 111 patients with lymphedema, including 96 limbs and 24 perineal areas affected. Three hundred forty-five lymphatics in the limbs and 52 in the perineum underwent anastomosis and were analyzed. Comparable lymphatic vessel diameter (inner: 0.5–0.9 mm; outer: 0.8–0.9 mm) and depth (9-10 mm) measurements across HFUS, CEUS, and combined HFUS + CEUS. However, HFUS + CEUS significantly improved detection sensitivity, identifying 313 vessels (91.1% accuracy) vs 114 (88.6%) for HFUS and 22 (90.9%) for CEUS. Significant postoperative reductions in limb circumference (39.3 ± 7.4 cm to 37.8 ± 7.1 cm) and volume (8.23 ± 3.63 L to 7.52 ± 3.39 L, *p* < 0.001). All ultrasound methods consistently showed volume reduction (HFUS: 9.37 ± 2.93 L to 8.46 ± 2.01 L; CEUS: 9.46 ± 2.57 L to 9.09 ± 2.45 L; HFUS + CEUS: 9.30 ± 3.21 L to 8.07 ± 3.12 L, *p* < 0.001–0.002).

**Conclusions:**

High-frequency US combined with CEUS serves as a reliable pre-op lymphatic mapping alternative when ICG lymphography fails.

**Key Points:**

***Question*** *In over 40% of lymphedema patients, preoperative ICG lymphangiography fails to show a linear pattern; can HFUS and CEUS provide complementary information?*

***Findings**** ICG failed to visualize in 42.53% of patients; HFUS and CEUS identified lymphatics in all and achieved 94.5% accuracy*.

***Clinical relevance**** This study confirmed that HFUS combined with CEUS improves the detection of lymphatic vessels and the success of LVA in ICG-negative cases*.

**Graphical Abstract:**

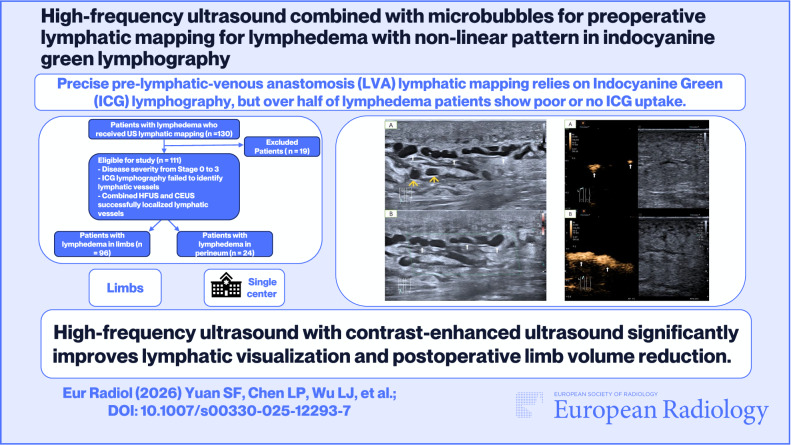

## Introduction

Lymphedema is the chronic accumulation of lymphatic fluid within the subcutaneous tissues due to the dysfunction of lymphatic delivery. Lymphedema may originate from congenital abnormalities or external damage to the lymphatic system, including lymph node removal surgery, radiation therapy, infections, or venous insufficiency. The increasing incidence of breast and gynecological cancers has led to a higher prevalence of lymphedema, highlighting the need for effective therapeutic strategies. Lymphatic-venous anastomosis (LVA), a microsurgical procedure that creates connections between lymphatic vessels and adjacent veins to facilitate lymphatic fluid drainage, is an effective treatment for lymphedema. It is characterized by favorable postoperative effects and minimal complications [1, 2]. Achieving successful outcomes of LVA requires accurate identification of well-functioning lymphatic vessels and subcutaneous veins with compatible diameters, which is challenging for surgeons.

Lymphatic vessel localization using indocyanine green (ICG) is a commonly used preoperative examination for LVA [3]. When ICG lymphography displays a linear pattern, lymphatic vessels can be accurately identified [4]. However, lymphatic vessels cannot be located on the stardust or diffuse pattern. ICG lymphography can only visualize lymphatic vessels at a depth of 20 mm subcutaneously, which often fails in patients with advanced edema complicated by severe fibrosis and fat hyperplasia [4]. According to a recent study, the detection rate of ICG lymphography is only 40% for patients with lower extremity lymphedema [5]. Patients who are allergic to iodine are also unable to be imaged using ICG [4, 6]. Therefore, novel imaging techniques as an alternative to ICG lymphography are required for the preoperative mapping of LVA.

Previous studies have reported the use of high-frequency ultrasound (HFUS) and contrast-enhanced ultrasound (CEUS) for lymphatic mapping. HFUS allows detailed imaging of subdermal veins and lymphatic vessels [7–9], as well as the visualization of lymphatic peristalsis. Ultrasound microbubbles are capable of accumulating in the lymphatic system following intradermal injection, enhancing the echogenicity of lymphatic vessels [10, 11]. If HFUS and CEUS were combined, they would enable the identification and mapping of lymphatic vessels, which would also allow for the measurement of vessel diameters and patency. This approach would be particularly valuable for assessing the function of lymphatic vessels, facilitating the selection of the optimal anastomosis sites for LVA. In this study, we utilized HFUS combined with CEUS for LVA mapping on patients with lymphedema, for whom the ICG mapping failed. The aim of the study is to develop an efficient and accurate strategy for targeting functional lymphatic vessels and compatible veins, serving as an alternative to ICG lymphography.

## Materials and methods

### Patients and study design

**Fig. 1:**
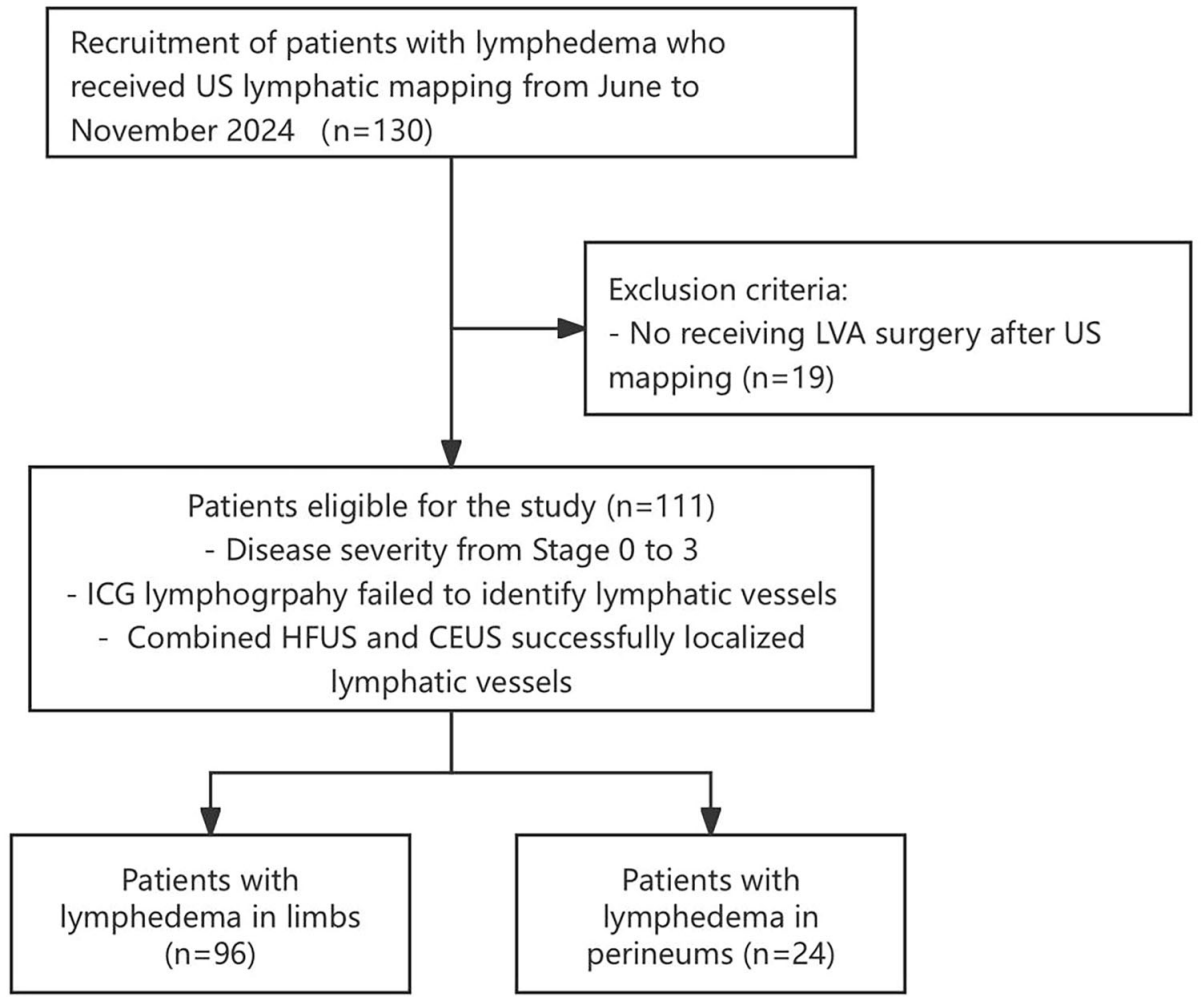
Schematic of the study flow. LVA, lymphatic-venous anastomosis; ICG, indocyanine green; HFUS, high-frequency ultrasound; CEUS, contrast-enhanced ultrasound

The study was designed as a single-center retrospective study. All patients who underwent preoperative HFUS and CEUS lymphatic mapping for LVA surgery at Peking University Shenzhen Hospital from June to November 2024 were included (Fig. 1). The inclusion criteria were as follows: (1) patients with lymphedema whose disease severity was classified from stage 0 to 3 at the time of the lymphatic ultrasound, according to the International Society for Lymphology criteria [1]; (2) ICG lymphography failed to visualize the linear pattern; and (3) lymphatic vessels and subcutaneous veins were successfully identified by HFUS and CEUS before surgery. Exclusion criteria included patients who did not receive LVA surgery after mapping. This study was approved by the ethics committee of Peking University Shenzhen Hospital (No. [2025023]). All patients provided written informed consent for participation.

### ICG fluorescent lymphography

The ICG fluorescent lymphography was performed prior to the US examination. A plastic surgeon with over 5 years of experience conducted the procedure, and another surgeon with more than 10 years of experience assessed the results. A total volume of 0.1 mL ICG (0.5%, Dandong Yichuang Pharmaceutical Co., Ltd) was injected subcutaneously into all interdigital spaces and variable locations of the affected limb. A gentle massage was applied to facilitate the diffusion and flow of the injected ICG. The images were classified according to the Maegawa classification [4], patterned by the plastic surgeon.

### Preoperative lymphatic ultrasound

Three radiologists (Dr. Yuan, with 20 years of experience in ultrasound diagnostics, and Dr. Zhao, with 9 years of experience in ultrasound diagnostics) performed the lymphatic mapping via HFUS and CEUS for the patients. The ultrasound images were preserved in both static and dynamic formats, subsequently evaluated by the two radiologists. If there was a discrepancy in image interpretation among the two radiologists, a senior doctor (Dr. Hu with 24 years of experience in ultrasound diagnostics) determined the final result. They were not blinded to the ICG results. We assessed inter-rater reliability for the categorical ratings using Fleiss’ kappa (κ). Given *m* = 3 independent raters assessing *N* = 503 items across *k* = 2 categories, Fleiss’ kappa estimates the agreement beyond chance. We reported κ with 95% confidence intervals (CI) and *p*-values, and interpreted the strength of agreement following Landis and Koch (1977): < 0.00 poor, 0.00–0.20 slight, 0.21–0.40 fair, 0.41–0.60 moderate, 0.61–0.80 substantial, 0.81–1.00 almost perfect. The Aplio i900 ultrasound machine (Canon Medical system) with two linear array transducers (i24LX8 [8–24 MHz] and i18LX5 [5–18 MHz]) was used for the US examination.

### HFUS procedure

The i24LX8 (frequency 8–24 MHz) transducer was used for HFUS. The depth was 2–2.5 cm, and the focus point was set at 1 cm. The radiologists searched for the lymphatic vessels beneath the superficial fascia on the transverse section. The inner and outer diameters of the vessel were measured on the longitudinal section. And the longitudinal courses of the vessels were marked on the skin. Within 1 cm of the marked lymphatic vessels, the adjacent subcutaneous vein of similar size was further identified using SMI (Superb Micro-vascular Imaging) mode and marked on the skin.

### CEUS procedure

The i18LX5 probe was used for CEUS, with a mechanical index of 0.08. The injection points of the upper limbs were web spaces of the fingers, the midpoint of the wrist striae, the radial side, the medial and lateral sides of the elbow, and the middle of the elbow. The lower extremities were injected at the web spaces of the toes, medial malleolus, and medial and lateral of the knee. 0.3 mL of SonoVue suspension (Bracco, lyophilized powder SF6 microbubbles 59 mg) was injected intracutaneously using a 21G needle at each injection point, and then gently massaged the surrounding tissue. The highly enhanced tubular structures were identified as lymphatic vessels, which were subsequently measured by HFUS and marked on the skin.

### HFUS and CEUS image criteria

**Fig. 2:**
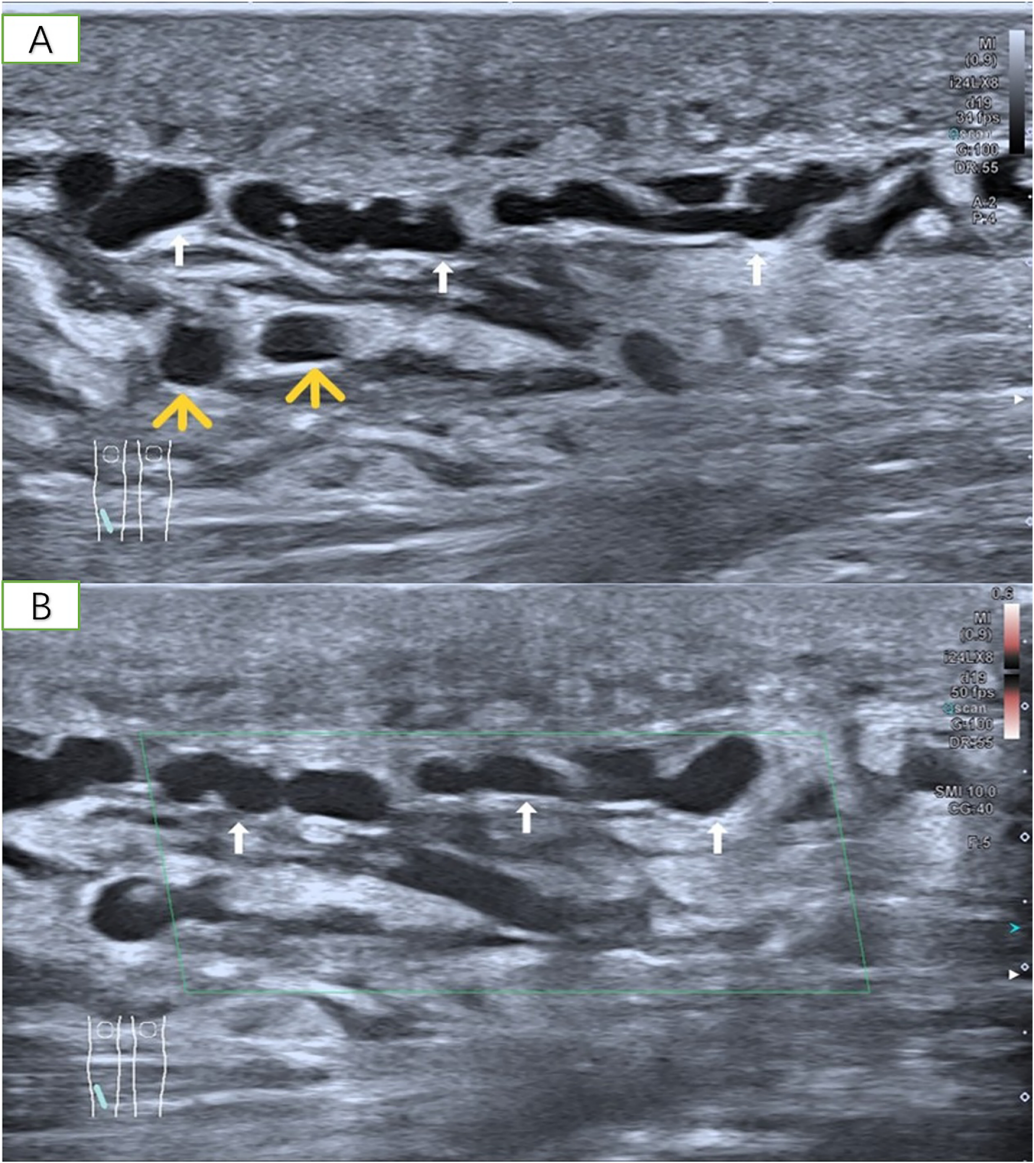
**A** HFUS indicates lymphatic vessel (yellow arrow) is a structure with a circumferential annular hyperechoic and central anechoic structure on the transverse section, and parallel hyperechoic linear wall structures accompanied by anechoic lumens in the longitudinal section (white arrow). **B** SMI indicates no obvious blood flow signal in the lumen of the lymphatic vessel. HFUS, high-frequency ultrasound; SMI, Superb Micro-vascular imaging

HFUS criteria for identifying lymphatics: (a) In the transverse section, a structure with a circumferential annular hyperechoic and central anechoic structure in the superficial fascia layer [12], while on the longitudinal section, it is parallel hyperechoic linear wall structures accompanied by anechoic lumens. These tubular structures may converge with other vessels or give off branches. (b) SMI: no obvious blood flow signal detected within the lumen (Fig. 2). (c) After squeezing and stimulation, some anechoic ducts can have diastolic and contractile movements, that is, lymphatic peristalsis (Video 1, Electronic Supplemental Material).

**Fig. 3:**
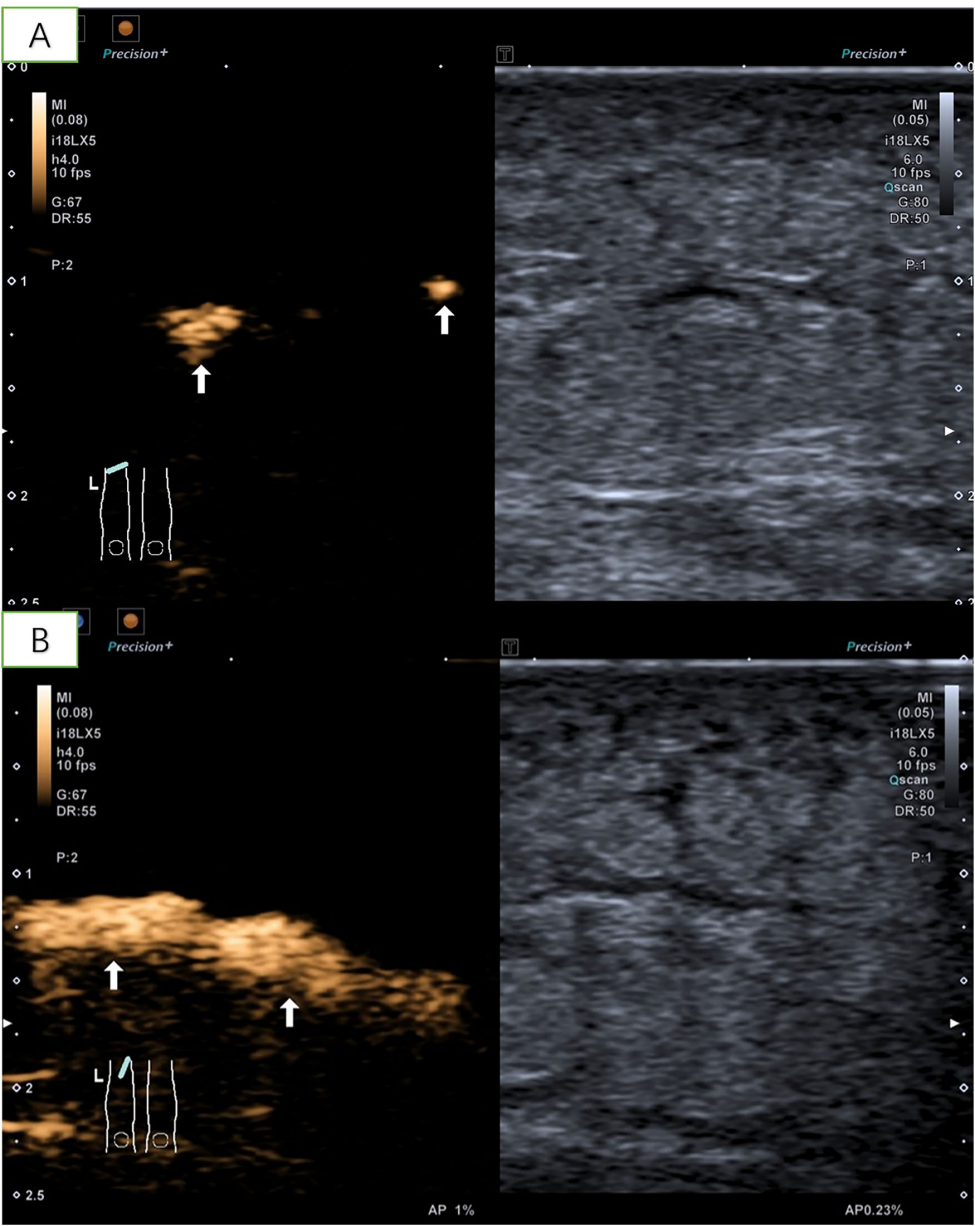
**A** CEUS shows that lymphatic vessels are round-like structures that appear in the superficial fascia layer in the transverse section. **B** CEUS revealed linear hyperenhancement with contrast agent bubbles accumulating inside the lumen of the lymphatic vessel along the axis. (white arrow). CEUS, contrast-enhanced ultrasound

CEUS image criteria to determine lymphatics: (a) In the transverse section, single or multiple spotted or round-like heights appear in the superficial fascia layer, which can be continuously traced along the flow direction and may converge or branch with peripheral spotted enhancement. (b) On the longitudinal section, linear hyperenhancement with visible contrast agent bubbles flowing along the axis (Fig. 3).

### Intraoperative confirmation of successful anastomosis

ICG fluorescence imaging: visible passage of ICG dye from lymphatic vessels into the vein under near-infrared imaging [13].

### Measurement

Lymphatic measurement: inner diameter, outer diameter, and distance from the skin surface; venous measurement: inner diameter and distance from the skin surface.

### Limb circumference measurement

The circumferences of the edematous limbs were measured by a microsurgeon with five years of experience prior to the imaging examinations for all patients. For measuring the lower limb circumference, the ankle joint was used as the starting point, and the limb circumference was measured every 5 cm up to the root of the thigh. For the upper limb circumference, the wrist joint was used as the starting point, and measurements were taken every 5 cm up to the root of the upper arm. Using the same method, the limb circumference was measured again within one week after LVA surgery by the same surgeon, and the limb volume was calculated according to a formula [14].

### Statistical analysis

All patients underwent LVA surgery within one week after ultrasonography, and limb circumference was measured for limb volume calculation [14] after surgery within one week. The primary endpoint was intraoperative lymphatic vessel localization accuracy, the number of lymphatic vessels, and the anastomosis success rate. The Kruskal-Wallis test was used to compare the differences among the three lymphatic ultrasound imaging modalities (HFUS, CEUS, and HFUS + CEUS) in terms of the number of detected lymphatic vessels, diameter, depth, and accuracy rate. The secondary endpoints were the affected limbs’ volume and circumference in a week post-operation compared with that pre-operation, calculated by paired *T*-test. Pairwise paired *T*-tests were used to compare postoperative volume differences among HFUS, CEUS, and HFUS + CEUS lymphatic imaging methods. SPSS 25.0 statistical software was used for analysis. Normally distributed data are expressed as mean ± SD; and Statistical significance was set at *p* < 0.05.

## Results

The schematic of the study flow is illustrated in Fig. 1. From June to November 2024, 261 patients with 313 sites (limbs and perineum) underwent preoperative ICG lymphography and LVA surgery, among which 111 patients (42.53%) and 120 sites (96 limbs and 24 perineum, 38.33%) were included in this study. Of the 111 patients, 12 had primary lymphedema, and 99 had secondary lymphedema. Among the patients with primary lymphedema, 2 were diagnosed with Klippel-Trenaunay Syndrome and 3 were diagnosed with congenital malformations of lymphatic vessels, and the remaining causes were unknown. Among patients with lymphedema secondary to malignant tumor surgery, 61 were afflicted with cervical cancer, 14 with breast cancer, 14 with endometrial cancer, 3 with ovarian cancer, 2 with penile cancer, 1 with Hodgkin’s lymphoma, 1 with lung adenocarcinoma, 1 with skin cancer, and 1 with gastric cancer. Additionally, one patient developed lymphedema following acupuncture treatment. The characteristics of the patient are detailed in Table 1.

**Table 1:** The characteristics of the patients

Characteristics	Values
Age(years)	53.79 ± 10.42 (24–79)
Gender	
Male	11
Female	100
Limbs	
Upper limbs	17
Lower limbs	79
Duration of edema (years)	5.85 (1 month–34 years)
International Society for Lymphology Stage (*n*%)	
Stage 1	0 (0%)
Stage 2	76 (63.3%)
Stage 3	44 (36.7%)
Mean limb circumference (cm)	
Upper limbs	28.52 ± 3.59
Lower limbs	40.83 ± 5.67
Mean subcutaneous soft tissue thickness of the perineum (mm)	28.63 ± 6.17

Agreement was almost perfect (κ = 0.9728; 95% CI, 0.9478–0.9913; *p* < 0.001) per Landis and Koch’s benchmarks (0.81–1.00). No severe adverse reactions were observed in patients who received lymphatic CEUS.HFUS + CEUS localized 503 lymphatic vessels. Three hundred ninety-seven skin incisions were made for LVA (100% success rate). Three hundred forty-five limb and 52 perineal anastomoses were performed, with preoperative lymphatic localization accuracy confirmed as 94.5% (326/345) and 71.8% (37/52), respectively. HFUS measured lymphatic vessels: inner diameter 0.51 ± 0.21 mm, outer diameter 0.92 ± 0.29 mm, depth 9.38 ± 3.78 mm. CEUS measured: inner diameter 0.53 ± 0.28 mm, outer diameter 0.86 ± 0.38 mm, depth 9.41 ± 3.56 mm (Table 2).

**Table 2:** Comparison of lymphatic vessel detection capabilities among different imaging methods

	HFUS	CEUS	HFUS + CEUS
Case count	29	7	60
Number of lymphatics	114	22	313
Anastomosed lymphatics	98	22	225
Accuracy (%)	88.6	90.9	91.1

*CEUS* contrast-enhanced ultrasound, *HFUS* high-frequency ultrasound

Comparative analysis of HFUS (*n* = 29, 114 vessels, 98 anastomoses, 88.6% accuracy), CEUS (*n* = 7, 22 vessels, 22 anastomoses, 90.9% accuracy), and HFUS + CEUS (*n* = 60, 313 vessels, 225 anastomoses, 91.1% accuracy) (Table 3), revealed HFUS + CEUS as optimal. *p*-values showed no significant differences: HFUS vs CEUS (*p* = 0.999), HFUS vs combined (*p* = 0.882), CEUS vs combined (*p* = 0.996).

**Table 3:** The lymphatic vessel detection results table for three different methods

Average (mm)	HFUS	CEUS	HFUS + CEUS
Inner diameter	0.538 ± 0.207	0.586 ± 0.277	0.517 ± 0.197
Outer diameter	0.821 ± 0.371	0.906 ± 0.378	0.829 ± 0.279
Depth	9.378 ± 3.778	9.888 ± 3.507	10.183 ± 4.18

*CEUS* contrast-enhanced ultrasound, *HFUS* high-frequency ultrasound

Quantitative analysis of lymphatic vessel dimensions by HFUS (inner/outer depth: 0.538 ± 0.207/0.821 ± 0.371/9.378 ± 3.778 mm), CEUS (0.586 ± 0.277/0.906 ± 0.378/9.888 ± 3.507 mm), and HFUS + CEUS (0.517 ± 0.197/0.829 ± 0.279/10.183 ± 4.180 mm) (Table 4). Statistical analysis of dimensional parameters yielded *p*-values of 0.983 (HFUS vs CEUS), 0.672 (HFUS vs HFUS + CEUS), and 0.820 (CEUS vs HFUS + CEUS), indicating no statistically significant differences among the three imaging modalities (*p* > 0.05 for all comparisons).

**Table 4:** Preoperative–postoperative limbs’ volume and circumference Paired *T*-test

		Average value		*p*-value
		Pre-operation	Post-operation	
Pair 1	Limb circumference (cm)	39.30 ± 7.40	37.84 ± 7.18	< 0.001
Pair 2	Preoperative-postoperative limb volume (L)	8.23 ± 3.63	7.52 ± 3.39	< 0.001

Sixty-four lymphatic vessels were identified in the perineal region. HFUS detected vessels in 20/24 cases (83.3%), 52 vessels (81.3%); CEUS: 4/24 cases (16.7%), 12 vessels (18.8%). HFUS + CEUS achieved 100% visualization (24/24 cases) and, overall accuracy of 71.88%.

Surgery significantly reduced limb circumference (39.30 ± 7.40 → 37.84 ± 7.18 cm, *p* < 0.001) and volume (8.23 ± 3.63 L → 7.52 ± 3.39 L, *p* < 0.001) (Table 5). Pre-op volumes: HFUS 9.37 ± 2.93 L, CEUS 9.46 ± 2.57 L, HFUS + CEUS 9.30 ± 3.21 L; post-op: HFUS 8.46 ± 2.01 L, CEUS 9.09 ± 2.45 L, HFUS + CEUS 8.07 ± 3.12 L (Table 5). All methods showed significant volume reduction (HFUS *p* = 0.001, CEUS *p* < 0.001, HFUS + CEUS *p* = 0.002). There were no significant differences among the three groups (HFUS vs CEUS *p* = 0.661, HFUS vs HFUS + CEUS *p* = 0.135, CEUS vs HFUS + CEUS *p* = 1.000).

**Table 5:** Paired *T*-test for postoperative follow-up volume differences in patients using three lymphatic vessel ultrasound detection methods

Limb volume (L)	HFUS	CEUS	HFUS + CEUS
Pre-operation	9.37 ± 2.93	9.46 ± 2.57	9.30 ± 3.21
Post-operation	8.46 ± 2.01	9.09 ± 2.45	8.07 ± 3.12
*p*-value	0.001	<0.001	0.002

*CEUS* contrast-enhanced ultrasound, *HFUS* high-frequency ultrasound

## Discussion

Recently, the application of ultramicroscopic technology in LVA surgery for patients with lymphedema has shown good results [15, 16], which is often guided by ICG lymphography. For patients in whom ICG lymphography fails to visualize lymphatic vessels, it is difficult for the surgeons to identify suitable lymphatic vessels for LVA [17]. Hence, it is necessary to find a reliable preoperative alternative to lymphography for these patients with no satisfying results of ICG lymphography. In this study, we aim to explore the feasibility of combined HFUS and CEUS in lymphatic mapping and their potential role as an alternative approach to ICG lymphography. We enrolled 111 patients with lymphedema, comprising 96 limbs and 24 perineal regions. Combined HFUS and CEUS localized 503 lymphatic vessels preoperatively, with 345 limb and 52 perineal incisions analyzed. HFUS detected vessels (0.51 ± 0.21 mm inner diameter, 0.92 ± 0.29 mm outer diameter, 9.3 ± 3.78 mm depth), CEUS (0.53 ± 0.28/0.86 ± 0.38/9.41 ± 3.56 mm). Surgical accuracy: 94.5% (limbs) and 71.8% (perineum). Postoperative limb circumference/volume reduced significantly (*p* < 0.001). HFUS + CEUS effectively guides LVA when ICG fails.

HFUS is the most commonly used imaging modality for many diseases, due to its ease of operation, low cost, good reproducibility, and no risk of allergies [18]. Recent 24/33 MHz HFUS probes enable precise lymphatic vessel imaging, including lumen/wall visualization and diameter/depth measurements [19]. HFUS assists the microsurgeons, especially those less experienced in selecting larger, superficial lymphatics and marking anastomosis veins, improving quality and efficiency [20]. CEUS clearly visualizes lymphatic pathways and transport function with minimal allergic risks [21]. Ultrasound contrast agents are metabolized by the breath and can also be used in patients with liver and kidney insufficiency, which have a wide range of clinical applications [22]. Consistent measurements across techniques confirmed comparable accuracy for preoperative lymphatic assessment in this study, supporting their clinical utility for preoperative planning in lymphatic microsurgery. For severe edema cases where HFUS/CEUS fail, HFUS can mark superficial veins (e.g., saphenous, cephalic) to expedite intraoperative lymphatic localization [23, 24]. Previous studies have demonstrated that the use of HFUS and CEUS significantly enhances the precision of identifying lymphatic vessels and veins during LVA surgery, thereby reducing the overall surgical time for patients. Hara et al [7] reported the effectiveness of US in lymphatic mapping for LVA surgery. Jang et al [10] also validated that CEUS could identify lymphatic vessels when ICG lymphography failed in 11 cases. This study included more ICG-failed cases successfully mapped by HFUS + CEUS. Although HFUS or CEUS alone achieved > 88% accuracy, their combination significantly improved lymphatic vessel detection and anastomosis success rates, suggesting HFUS + CEUS as an optimal preoperative imaging strategy. These findings confirm the value of HFUS and CEUS in pre-LVA lymphatic mapping, especially for ICG-negative cases. HFUS demonstrates a higher detection rate of lymphatic vessels in patients with early-stage edema due to its high resolution. However, in patients with more severe edema, the presence of extensive soft tissue fibrosis and interstitial fluid accumulation creates complex ultrasound reflection interfaces, making it difficult to distinguish lymphatic vessels. In contrast, once the contrast agent enters the lymphatic lumen, it forms a clear contrast with the surrounding soft tissues. Therefore, CEUS not only improves the ultrasound detection rate of lymphatic vessels but also accelerates the detection process. Our study shows contrast-enhanced methods (CEUS and combined approach) better detect lymphatic changes linked to volume reduction than HFUS.

However, this study has several limitations: (1) being a single-center retrospective study, we plan to conduct a multicenter prospective study for validation; (2) patients came from across China, making monthly follow-ups difficult—we only measured postoperative limb circumference at discharge; and (3) perineal privacy concerns limited standardized edema assessment and increased dropout rates, requiring future improvement.

## Conclusion

Combined HFUS and CEUS can be applied as a novel and reliable preoperative lymphatic mapping method for patients with lymphedema, for whom the lymphatic vessels cannot be identified by ICG lymphography.

## Supplementary information

Video 1

ELECTRONIC SUPPLEMENTARY MATERIAL

## Abbreviations

CEUS: Contrast-enhanced ultrasound
HFUS: High-frequency ultrasound
ICG: Indocyanine green
LVA: Lymphatic-venous anastomosis
SMI: Superb Micro-vascular imaging
