# Correction: High-frequency ultrasound combined with microbubbles for preoperative lymphatic mapping for lymphedema with a non-linear pattern in indocyanine green lymphography

**DOI:** 10.1007/s00330-026-12509-4

**Published:** 2026-05-06

**Authors:** ShuFang Yuan, LiPing Chen, LanJing Wu, ChenYang Zhao, JingJing WEN, Long Biao YU, ZheGang Zhou, DeSheng Sun, ZhengMing Hu

**Affiliations:** 1 Department of Ultrasound Imaging, Peking University Shenzhen Hospital, Shenzhen, China; 2 Medical Technology Department, The Islands Healthcare Complex—Macao Medical Center of Peking Union Medical College Hospital, Shenzhen, China; 3 Microsurgery & Lymphatic Surgery Department, Peking University Shenzhen Hospital, Shenzhen, China

**Correction to: European Radiology**

10.1007/s00330-025-12293-7 published online 24 January 2026

In the original article, findings in the “Abstract” and “Results” sections were inaccurate and were corrected.

**Abstract/Results**

The sentence starting with “Significant postoperative reductions …” incorrectly stated “volume (81.1 ± 35.8 L to 74.2 ± 33.4 L, *p* < 0.001)” and was corrected to “volume (8.23 ± 3.63 L to 7.52 ± 3.39 L, *p* < 0.001)”.

The sentence “All ultrasound methods consistently showed volume reduction (HFUS: 93.3 ± 25.4 L to 89.6 ± 24.1 L; CEUS: 92.4 ± 28.9 L to 83.4 ± 19.8 L; HFUS + CEUS: 91.2 ± 31.8 L to 84.2 ± 21.5 L, *p* < 0.001–0.002)” was corrected to “All ultrasound methods consistently showed volume reduction (HFUS: 9.37 ± 2.93 L to 8.46 ± 2.01 L; CEUS: 9.46 ± 2.57 L to 9.09 ± 2.45 L; HFUS + CEUS: 9.30 ± 3.21 L to 8.07 ± 3.12 L, *p* < 0.001–0.002)”.

**Results**

In the sentence starting with “Surgery significantly reduced limb circumference …” incorrectly stated “volume (81.15 ± 35.84→74.19 ± 33.44 L, *p* < 0.001) (Table 5). Pre-op volumes: HFUS 93.27 ± 25.38 L, CEUS 92.37 ± 28.91 L, HFUS + CEUS 91.15 ± 31.75 L; post-op: HFUS 89.60 ± 24.13 L, CEUS 83.43 ± 19.78 L, HFUS + CEUS 84.19 ± 21.47 L (Table 5)” and was corrected to “volume (8.23 ± 3.63 L → 7.52 ± 3.39 L, *p* < 0.001) (Table 5). Pre-op volumes: HFUS 9.37 ± 2.93 L, CEUS 9.46 ± 2.57 L, HFUS + CEUS 9.30 ± 3.21 L; post-op: HFUS 8.46 ± 2.01 L, CEUS 9.09 ± 2.45 L, HFUS + CEUS 8.07 ± 3.12 L (Table 5).”

In the same paragraph, the following sentence incorrectly stated “All methods showed significant volume reduction (HFUS *p* < 0.001, CEUS *p* = 0.002, HFUS + CEUS *p* = 0.001)” and was corrected to “All methods showed significant volume reduction (HFUS *p* = 0.001, CEUS *p* < 0.001, HFUS + CEUS *p* = 0.002)”.

**Furthermore, Tables 4 and 5 were corrected:**

In Table 4, the values in row “Pair 2” incorrectly stated:

**Table:** 

Pair 2	Preoperative-postoperative limb volume (L)	81.15 ± 35.84	74.19 ± 33.44	< 0.001

The revised Table 4 can be seen below:

**Table 4 Preoperative-postoperative limbs’ volume and circumference paired T-test**

**Table:** 

		Average value		*p*-value
		Pre-operation	Post-operation	
Pair 1	Limb circumference (cm)	39.30 ± 7.40	37.84 ± 7.18	< 0.001
Pair 2	Preoperative-postoperative limb volume (L)	8.23 ± 3.63	7.52 ± 3.39	< 0.001

Table 5 incorrectly stated:

**Table 5 Paired T-test for postoperative follow-up volume differences in patients using three lymphatic vessel ultrasound detection methods**

**Table:** 

Limb volume (L)	HFUS	CEUS	HFUS + CEUS
Pre-operation	93.27 ± 25.38	92.37 ± 28.91	91.15 ± 31.75
Post-operation	89.60 ± 24.13	83.43 ± 19.78	84.19 ± 21.47
*p*-value	< 0.001	0.002	0.001

The revised Table 5 can be seen below:

**Table 5 Paired T-test for postoperative follow-up volume differences in patients using three lymphatic vessel ultrasound detection methods**

**Table:** 

Limb volume (L)	HFUS	CEUS	HFUS + CEUS
Pre-operation	9.37 ± 2.93	9.46 ± 2.57	9.30 ± 3.21
Post-operation	8.46 ± 2.01	9.09 ± 2.45	8.07 ± 3.12
*p*-value	0.001	< 0.001	0.002

The original article has been corrected.

