# Supplementary material for: High-frequency ultrasound combined with microbubbles for preoperative lymphatic mapping for lymphedema with a non-linear pattern in indocyanine green lymphography
Source: Eur Radiol. 2026 Jan 24;36(7):5243–52. doi: 10.1007/s00330-025-12293-7 (PMC13282317; doi:10.1007/s00330-025-12293-7)
Supplement: Supplementary file 2 — ELECTRONIC SUPPLEMENTARY MATERIAL [file 330_2025_12293_MOESM2_ESM.docx]

Video 1 The lymphatic vessel had diastolic and contractile movements while after stimulation.
